# Supplementary material for: Causal Relationship between Aging and Anorexia Nervosa: A White-Matter-Microstructure-Mediated Mendelian Randomization Analysis
Source: Biomedicines. 2024 Aug 16;12(8):1874. doi: 10.3390/biomedicines12081874 (PMC11351342; doi:10.3390/biomedicines12081874)
Supplement: Supplementary file 1 [file biomedicines-12-01874-s001.zip › Supplementary Figure/Figure S4/Singe SNP plot of MR analysis for TL to ubm-b-1539.pdf]

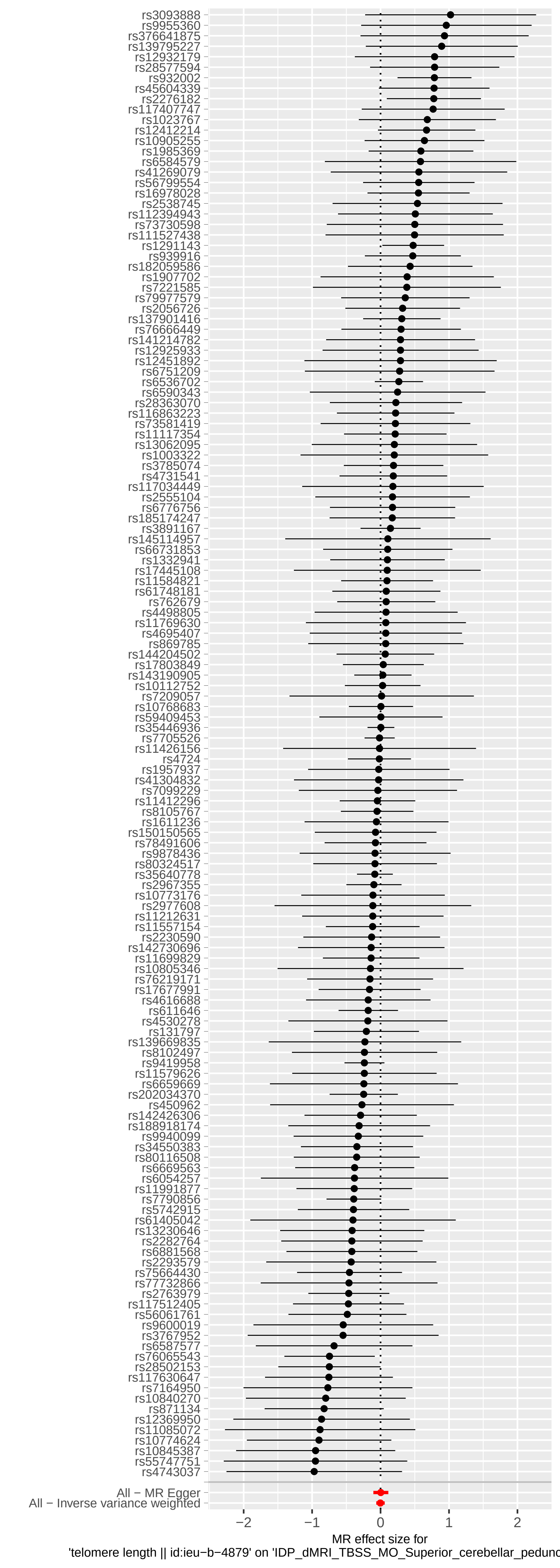
rs3093888
rs9955360
rs376641875
rs139795227
rs12932179
rs28577594
rs932002
rs45604339
rs2276182
rs117407747
rs1023767
rs12412214
rs10905255
rs1985369
rs6584579
rs41269079
rs56799554
rs16978028
rs2538745
rs112394943
rs73730598
rs111527438
rs1291143
rs939916
rs182059586
rs1907702
rs7221585
rs79977579
rs2056726
rs137901416
rs76666449
rs141214782
rs12925933
rs12451892
rs6751209
rs6536702
rs6590343
rs28363070
rs116863223
rs73581419
rs11117354
rs13062095
rs1003322
rs3785074
rs4731541
rs117034449
rs2555104
rs6776756
rs185174247
rs3891167
rs145114957
rs66731853
rs1332941
rs17445108
rs11584821
rs61748181
rs762679
rs4498805
rs11769630
rs4695407
rs869785
rs144204502
rs17803849
rs143190905
rs10112752
rs7209057
rs10768683
rs59409453
rs35446936
rs7705526
rs11426156
rs4724
rs1957937
rs41304832
rs7099229
rs11412296
rs8105767
rs1611236
rs150150565
rs78491606
rs9878436
rs80324517
rs35640778
rs2967355
rs10773176
rs2977608
rs11212631
rs11557154
rs2230590
rs142730696
rs11699829
rs10805346
rs76219171
rs17677991
rs4616688
rs611646
rs4530278
rs131797
rs139669835
rs8102497
rs9419958
rs11579626
rs6659669
rs202034370
rs450962
rs142426306
rs188918174
rs9940099
rs34550383
rs80116508
rs6669563
rs6054257
rs11991877
rs7790856
rs5742915
rs61405042
rs13230646
rs2282764
rs6881568
rs2293579
rs75664430
rs77732866
rs2763979
rs117512405
rs56061761
rs9600019
rs3767952
rs6587577
rs76065543
rs28502153
rs117630647
rs7164950
rs10840270
rs871134
rs12369950
rs11085072
rs10774624
rs10845387
rs55747751
rs4743037
All − MR Egger
All − Inverse variance weighted
−2
−1
0
1
2
MR effect size for
'telomere length || id:ieu−b−4879' on 'IDP_dMRI_TBSS_MO_Superior_cerebellar_peduncl
